# Presence of thallium in the environment: sources of contaminations, distribution and monitoring methods

**DOI:** 10.1007/s10661-016-5647-y

**Published:** 2016-10-26

**Authors:** Bozena Karbowska

**Affiliations:** Institute of Chemistry and Technical Electrochemistry, Poznan University of Technology, ul. Berdychowo 4, 61-138 Poznan, Poland

**Keywords:** Thallium, Toxicity, Laser excited atomic fluorescence spectrometry (LEAFS), Inductively coupled plasma mass spectrometry (ICP-MS), High-resolution inductively coupled plasma mass spectrometry (HR-ICP-MS), Electrothermal vaporization inductively coupled plasma mass spectrometry (ETV-ICP-MS), Atomic absorption spectrometry (FAAS and GFAAS), Zeeman effect electrothermal absorption spectrometry (ZEETAS), Differential pulse anodic stripping voltammetry (DPASV)

## Abstract

Thallium is released into the biosphere from both natural and anthropogenic sources. It is generally present in the environment at low levels; however, human activity has greatly increased its content. Atmospheric emission and deposition from industrial sources have resulted in increased concentrations of thallium in the vicinity of mineral smelters and coal-burning facilities. Increased levels of thallium are found in vegetables, fruit and farm animals. Thallium is toxic even at very low concentrations and tends to accumulate in the environment once it enters the food chain. Thallium and thallium-based compounds exhibit higher water solubility compared to other heavy metals. They are therefore also more mobile (e.g. in soil), generally more bioavailable and tend to bioaccumulate in living organisms. The main aim of this review was to summarize the recent data regarding the actual level of thallium content in environmental niches and to elucidate the most significant sources of thallium in the environment. The review also includes an overview of analytical methods, which are commonly applied for determination of thallium in fly ash originating from industrial combustion of coal, in surface and underground waters, in soils and sediments (including soil derived from different parent materials), in plant and animal tissues as well as in human organisms.

## Introduction

Contamination with heavy metals is considered as a major environmental issue and threat to human health. Thallium-based compounds exhibit a high tendency to accumulate in the environment. Alleviated level of thallium emissions as well as its deposition over time may lead to major environmental pollution. Prolonged presence of thallium in terrestrial, aerial and aquatic systems may notably increase the exposure risks.

Thallium is considered as toxic for human and animal organisms, microorganisms and plants (Nriagu 1998; Peter and Viraraghavan 2005; Kazantzis 2000). The toxicity of this element is higher compared to mercury, cadmium and lead (maximum admissible concentration at 0.1 mg mL^−1^) (Repetto et al. 1998; Peter and Viraraghavan, 2005). The toxicity of thallium-based compounds is mainly caused by the similarity between thallium (I) ions and potassium ions (Groesslova et al. 2015), which results in the disorder of potassium-associated metabolic processes due to thallium interference (Sager 1998; Wojtkowiak et al. 2016). Human exposure to thallium is mainly associated with the consumption of contaminated food or drinking water. Thallium rapidly enters the bloodstream and is transported across the whole organism, which leads to accumulation in bones, kidneys and the nervous system. In consequence, the functioning of several relevant enzymes is disrupted. Stomach and intestinal ulcers, alopecia and polyneuropathy are considered as classic syndromes of thallium poisoning. Other symptoms include astral disorders, insomnia, paralysis, loss of body mass, internal bleeding, myocardial injury and, in consequence, death (Peter and Viraraghavan 2005; Kazantzis 2000; Galván-Arzate and Santamaria 1998). Ingestion of more than 1.5 mg of thallium per 1 kg of body mass may be fatal. Recent studies also indicate that high levels of Tl may be associated with an increased risk of low birth weight (Xia et al. 2016). Due to the abovementioned reasons, the concentration of thallium in the environment should be strictly monitored.

The aim of this review was to integrate the results of literature reports regarding the contents of thallium in various environmental samples and to highlight the most common analytical methods, which are used for determination of thallium at trace levels. The obtained data was used for assessment of thallium concentrations, which may be valuable during research focused on new sources of contamination with this toxic metal.

## Distribution of thallium in the environment

The first part of this review is focused on the distribution of thallium in the environment. This section covers the possible emission sources, aspects associated with transport of thallium-based contaminants throughout different environmental niches as well as their uptake by living organisms.

**Fig. 1:**
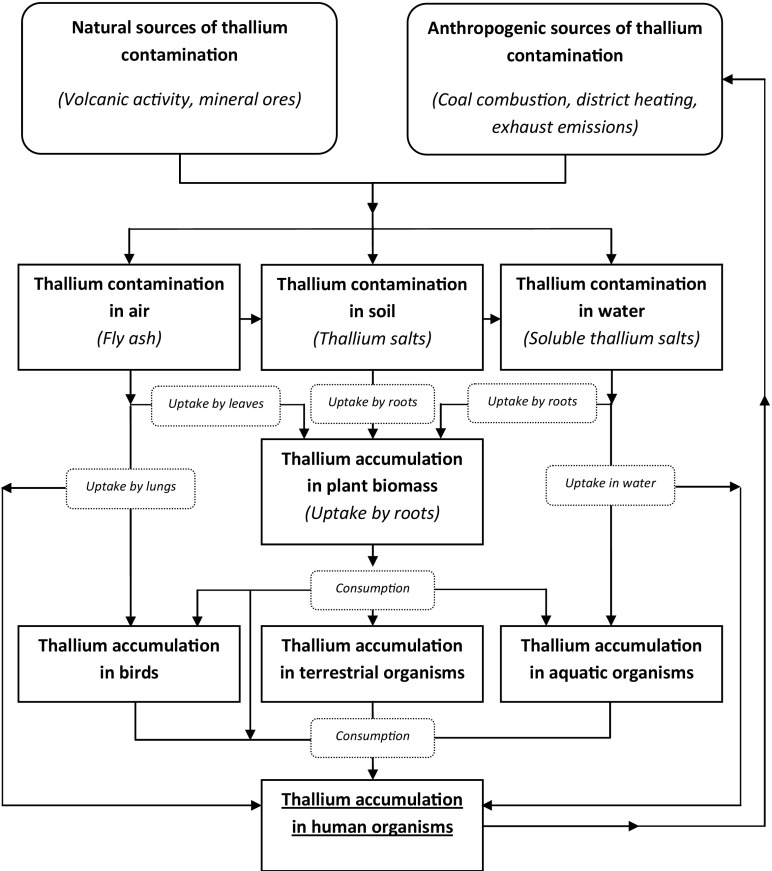
Idea scheme which presents the transport of thallium in the environment (based on Savariar 2014)

Thallium is naturally present in the environment, most notably in its terrestrial elements, although usually at low concentrations. Enrichment of specific niches with thallium-based compounds is a direct result of a specific transport pattern, which is shown in Fig. 1.

Emissions, which lead to increased concentrations of thallium in the environment, may be natural or associated with anthropogenic activity. The resulting air pollution (thallium ash) allows thallium contaminations to spread across wide distances and to enter other environmental niches as well as organisms (uptake by lungs). In the terrestrial environment, thallium is usually bound with the soil matrix, which considerably limits its transport, although dissolved thallium (soluble thallium salts) are susceptible to flushing and may be introduced to the aquatic environment. Such compounds may also leak into underground water streams and increase the risks of chronic exposure. On the other hand, high concentration of thallium in shallow soil also poses a notable threat due to possible uptake by plant roots and storage in plant biomass. As a result, thallium may enter the food chain and accumulate in living organisms, causing severe disorders and ultimately becoming fatal. In order to prevent thallium poisoning, its content must not exceed the environmentally safe limits, which are presented in Table 1.

**Table 1:** Environmentally safe limits for thallium

	Thallium limits	Sources
Drinking water	2 μg L^−1^	USEPA (2015), Xiao et al. (2004)
Arable soils	1 mg kg^−1^	CCME (2003), Xiao et al. (2004)
World land plants	0.008–1.0 mg kg^−1^	Kabata Pendias and Pendias (1992)
World edible plants	0.03–0.3 mg kg^−1^	
World average daily intake	2 μg day^−1^	Sabbioni et al. (1984), Xiao et al. (2004)
Oral reference dose	0.056 mg day^−1^	RAIS (2003), Xiao et al. (2004)

### Thallium in fly ash originating from industrial combustion of coal

The rapid technological and industrial expansion resulted in increased risk of environmental contamination with thallium. It is estimated that approx. 5000 t of thallium is released into the environment every year due to industrial activity (Dmowski et al. 2002), with approx. 1000 t originating from combustion of coal (Galván-Arzate and Santamaria 1998; Querol et al. 1995). A considerable amount of thallium is bound with sulphides (approx. 70 %), and the remaining content is bound with aluminosilicates and organic compounds (Querol et al. 1995). The products of coal combustion—slag and ash—contain higher concentrations of thallium. During combustion of coal and production of cement, thallium becomes oxidized at higher temperature and then condenses on the surface of ash particles, in low-temperature areas. Prolonged contact time between the ash and exhaust fumes leads to a notable concentration of thallium in fly ash, which is 2 to 10 times higher compared to the state prior to combustion (Cvjetko et al. 2010; Finkelman 1999). Global resources of thallium in coal amount to 630,000 t. In 2004, the production of thallium reached 12 t; however as of the beginning of 2005, it remains at a relatively stable level of 10 t per annum (Finkelman 1999).

The content of thallium in fly ash originating from a Polish cement plant ranged from 18 to 40 mg kg^−1^ (Kabata Pendias and Pendias 1999). The ash samples collected from filters installed in the chimneys of furnaces in the mining-metallurgical plant ‘Bolesław’ contained an average of 882 mg kg^−1^ of thallium (ash originating from a rotary furnace filter) and up to 5 % of thallium in ash from a sintering furnace (Kicińska 2009).

Furthermore, thallium is emitted to the atmosphere in the form of dust, vapours or liquids during industrial processing. This statement is consistent with the results of studies regarding the content of thallium in aerosols near Katowice city (Poland), as well as the amount of thallium determined in ashes and soils originating from national parks located at southern Poland. The content of thallium in aerosols from the downtown district of Katowice city was at 66 μg m^−3^; however, the value was much higher in the vicinity of metal smelters (Manecki et al. 1988; Schejbal-Chwastek and Tarkowski 1988; Tomza 1987). It should be noted that the amount of thallium in air cannot exceed the limit of 0.1 mg m^−3^ (OSHA 2015).

### Thallium in surface and underground waters

The content of thallium in river waters in Poland ranged from 5 to 17 ng L^−1^. Sea waters usually contain 10–15 ng L^−1^ of thallium (Lukaszewski et al. 1996; Lis et al. 2003; Kabata Pendias and Pendias 1999; Małuszyński 2009). Analysis of water samples collected from Ohrid Lake near Labino (Macedonia coordinates 41° 07′ 01″ N, 20° 48′ 06″ E) revealed that the concentration of thallium was at 0.5 μg L^−1^, whereas samples collected near Ljubanista contained approx. 0.3 μg L^−1^ (Stafilov and Cundeva 1998). The concentration of thallium in tap water from Skopje (Macedonia) amounted to 0.18 μg L^−1^. The contaminated waters of Huron and Raisin rivers in Michigan contained 21 and 2621 ng L^−1^ of thallium, accordingly (Garbai et al. 2000; Karlsson 2006). The water samples collected from the Silesian-Cracow region (Poland) were characterized by a higher thallium content, which ranged from 0.16 to 3.24 μg L^−1^ (Paulo et al. 2002)*.* Compared to the average thallium content in three main rivers in Poland, the concentration of thallium in the analyzed samples was two to three times higher (Lukaszewski et al. 1996). This suggests that thallium is released from river sediments.

The content of thallium in underground water samples typically amounts to 20–24 μg L^−1^; however, in deep groundwater samples, it ranges from 13 to 1100 μg L^−1^. The contents of thallium dissolved in groundwater samples from the Miocene level collected near Poznan city (western Poland) ranged from 0.005 to 0.47 μg L^−1^. The samples were collected from layers of fine-grained sand and silt divided by layers of brown coals. The concentration of thallium present in Warta River varied from 0.15 to 0.47 μg L^−1^. Total content of thallium in underground water samples ranged from 0.24 to 26 μg L^−1^. In case of suspensions of the underground brown water, the median amount of thallium reached 40–60 μg g^−1^ (Wojtkowiak et al. 2016; Lukaszewski et al. 2010).

Samples of water, bottom sediment and floodplain terrace soil of the Wodna and Luszowka rivulets, located in Trzebinia (Poland), were investigated in terms of thallium concentration. The obtained results are shown in Table 2. It can be observed that the thallium concentration in the water of both rivulets is significantly higher (20–30 times) than a typical level in surface water in Poland (Lukaszewski et al. 2010).

**Table 2:** Thallium concentration in bottom sediments, floodplain soil and water of the Wodna and Luszowka rivulets (Lukaszewski et al. 2010)

Level (m)	Thallium concentration (mg g^−1^ or mg L^−1^)	SD (mg g^−1^ or mg L^−1^)
Wodna rivulet		
Bottom sediment	7.4	0.7
Floodplain soil		
0–0.2	1.0	0.1
0.4–0.6	1.9	0.3
0.8–1.0	1.8	0.04
Rivulet water	0.32	0.04
Luszowka rivulet		
Bottom sediment	1.7	0.3
Floodplain soil		
0–0.2	0.11	0.03
0.4–0.6	0.20	0.05
0.8–1.0	0.08	0.01
Rivulet water	0.21	0.01

### Thallium in soils and sediments

Thallium participates in the formation of certain minerals, such as lorandite TlAsS_2_ (59 % Tl), hutchinsonite (PbTlAs_5_S_9_), crookesite (Cu, Tl, Ag)_2_Se (17 % Tl), urbaite TlAs_2_SbS_5_ (30 % Tl) or thallium (I) sulphide. Poorly soluble Tl_2_S is strongly bound by clay materials, manganese or iron compounds, including pyrites (Małuszyński 2009; Kabata Pendias and Pendias, 1999; Galván-Arzate and Santamaria 1998). High thallium content may be found in meteorites, granite and volcano rocks. It is ranked 67 in terms of distribution in the earth crust (amounting to approx. 3 × 10^−4^ of total mass %). The concentration of thallium in the lithosphere ranges from 0.3 to 0.6 mg kg^−1^ (Kabata Pendias and Pendias 1999). Thallium content in soils is strictly associated with the presence of thallium (I) ions in source rocks, which were formed due to soil-forming processes. Thallium in hydrothermal systems is bound with sulphides, such as pyrite, sphalerite or marcasite. The ventilation of such sulphides results in the dissemination of thallium in sedimentary rocks and organic compounds.

The concentration of thallium in igneous rocks ranges from 0.05 to 1.7 mg kg^−1^ (Lin and Nriagu 1998). Notably higher thallium content, ranging from 1.7 to 55 mg kg^−1^, was found in soils formed from limestone, marl or granite (Tremel et al. 1997). Extreme contents of thallium at a level of 1000 mg kg^−1^ were found in organic slates and carbon originating from the Jurassic period (Yang et al. 2005).

High concentration of this metal is associated with thallium-based sulfur salts: jordanite, gratonite, dufrenoisite and sphalerites (Vanek et al. 2015). These minerals were found in the ores from the mine in Bytom (Harańczyk 1965). In sphalerites, the concentrations of thallium amount to 500 mg kg^−1^ (Górecka 1996). In the Pomerania ores, the concentration of thallium may reach from 100 to 1000 mg kg^−1^ in dark, colloidal variations of sphalerites (Viets et al. 1996; Mayer and Sass-Gustkiewicz, 1998). In marcasite ores, the content of thallium may even exceed 1000 mg kg^−1^. More than ten times lower concentrations of thallium (up to 90 mg kg^−1^) are found in sphaleritic variations of most commonly exploited ores of Zn-Pb-Fe. The concentration of this element ranging from 36 to 70 mg kg^−1^ was found in rich sphaleritic ores from the Pomaranian mine, in the area of Olkusz (Cabała 2009; Górecka et al. 1996).

The concentration of thallium in iron sulphides from the eastern part of the Silesian-Cracow region ranged from 80 to 10,000 mg kg^−1^, whereas in case of zinc sulphides, the concentration ranged from 60 to 280 mg kg^−1^. Higher thallium content was found in pyrites from the western part of Silesian-Cracow ores (800–1200 mg kg^−1^). Post-flotation wastes may contain up to 5000 mg kg^−1^ of thallium (Karbowska 2004). The studies conducted by Karbowska (2004) indicate that there is a strong geochemical correlation between thallium and iron sulphides, as confirmed for zinc–lead ores, which were formed 150–200 millions of years ago (Sutkowska 2005; Jacher-Śliwczyńska and Schneider 2004).

Karbowska described the study of 120 soil samples of different geological origin (from the upper and lower level) from the Silesian-Cracow delves of zinc–lead ores (Karbowska 2004). The content of thallium determined in the upper level was higher by approx. 65 % compared to the lower layer and ranged from 0.04 to 29.8 mg kg^−1^. The lower concentration of thallium was noted for soils originating from quaternary fluvioglacial sands and gravels, with an average content in the lower and higher level at 0.1 and 0.18 mg kg^−1^, accordingly. The highest thallium content was found in ore-bearing formations of limestone from the middle Trias, which ranged from 0.45 to 4.66 mg kg^−1^ for the lower level (Lis et al. 2003). Samples collected from sand, gravel and sludge originating from river terraces contained from 0.13 to 1.87 mg kg^−1^ of thallium. The concentration of thallium in lower clay levels ranged from 0.14 to 35.1 mg kg^−1^. Samples collected from quaternary loess rocks contained 0.20–1.03 mg kg^−1^ of thallium. Notably high concentrations of thallium with a median value of 22.9 mg kg^−1^ were determined in samples of topsoil from waste heaps.

**Fig. 2:**
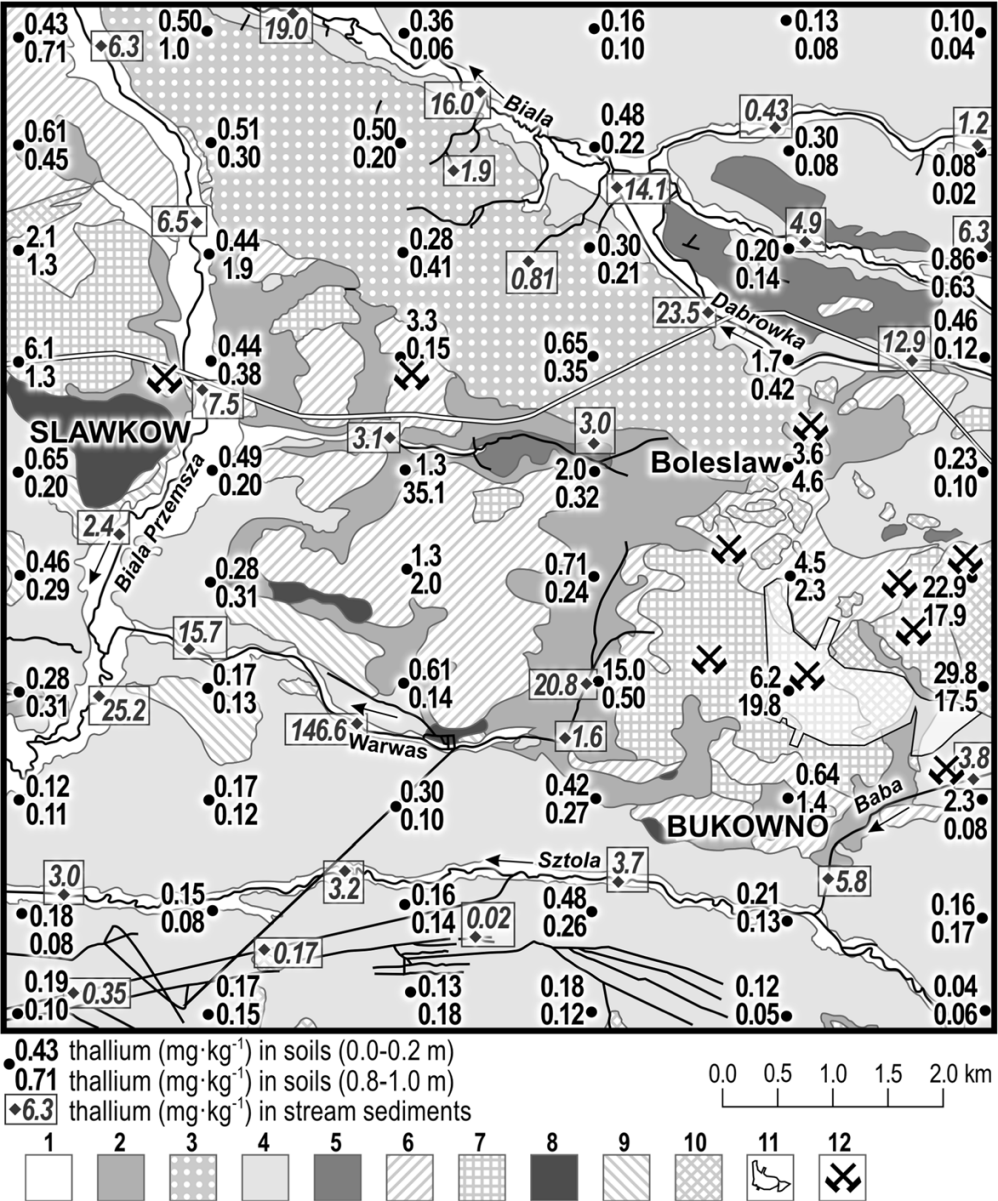
Content of total thallium (mg kg^−1^) in soils and sediments of the Silesian-Cracow delves of zinc–lead ores

Sludge samples from streams flowing through fluvioglacial sands and gravel in the southern part of the studied area were characterized by low thallium concentrations (0.02–0.17 mg kg^−1^). Bottom sediments from streams flowing though Pleistocene loess and formations from middle Trias, in the northern part of the area, contained 0.8–3.1 mg kg^−1^ of thallium. In case of bottom sediments originating from small rivers (Sztoła, Baba, Biała Przemsza, Dąbrówka), which often receive discharge streams from zinc–lead ore mines (including the Pomarian ore mine) as well as the local zinc smelter and flow through exposed ore-rich dolomites, the content of thallium reached 12.9 to 23.5 mg kg^−1^. The mean content of total thallium in soils and sediments of the Silesian-Cracow delves of zinc–lead ores is shown in Fig. 2 (Lis et al. 2003).

Thallium concentrations in the Lanmuchang area in southwestern Guizhou Province, China ranged from 100 to 35,000 mg kg^−1^ in sulphide ores and from 12 to 46 mg kg^−1^ in coals. Secondary minerals, produced by weathering, contain 25–1100 mg kg^−1^, with 32–2600 mg kg^−1^ in mine wasters. Altered host rocks contain 39–490 mg kg^−1^, with 6–330 mg kg^−1^ in outcropping rocks. Thallium levels in soils of the Lanmuchang area were at 40–124 mg kg^−1^ in soils from the mining area, 20–28 mg kg^−1^ in natural slope wash materials and 14–62 mg kg^−1^ in alluvial deposits downstream and ranged from 1.5 to 6.9 mg kg^−1^ in undisturbed natural soils (Xiao et al. 2004). Thallium concentrations in Korean soils near cement plants were distributed between 1.20 and 12.91 mg kg^−1^. However, soils near mines and smelters contained relatively low thallium concentrations ranging from 0.18 to 1.09 mg kg^−1^ (Jin-Ho Lee et al. 2015). Ores in western Małopolska contain up to 90 mg kg^−1^ (Zn sphalerite ores) and up to 1000 mg kg^−1^ of thallium (Fe marcasite ores) (Cabała 2009). High thallium concentrations were also found in soil from 100-year-old waste heap near Olkusz in Poland. Average thallium concentration reached 43 mg kg^−1^, while the highest thallium concentration equaled to 78 mg kg^−1^ (Wierzbicka et al. 2004). In turn, in Wiesloch (Germany), soils collected nearby a closed Pb and Zn mine contained 8.8–27.8 mg kg^−1^ (Schoer and Nagel 1980).

Soil samples collected from forest and grassland soils in Czech Republic by Vanek et al. (2009) contained 0.56–1.65 and 1.1–2.06 mg kg^−1^, respectively.

Due to the toxicity of thallium and its compounds, it is important to determine the concentration of this element in environmental and biological samples. Numerous researchers all over the world study this topic with the use of various analytical methods. Determination of the concentration of trace elements in different matrices requires the use of appropriate analytical techniques. The results of studies conducted by several authors are presented in Table 3. The following analytical techniques were used: laser excited atomic fluorescence spectrometry (LEAFS), inductively coupled plasma mass spectrometry (ICP-MS), differential pulse anodic stripping voltammetry (DPASV), high-resolution inductively coupled plasma mass spectrometry (HR-ICP-MS), electrothermal vaporization inductively coupled plasma mass spectrometry (ETV-ICP-MS) as well as atomic absorption spectrometry (flame atomic absorption spectrometry (FAAS) and graphite furnace atomic absorption spectrometry (GFAAS)). The analysis of the gathered data was conducted with respect to the type of the studied material, the sampling site, the employed analytical technique and the determined concentrations of thallium.

**Table 3:** Comparison of results of thallium content determination in various environmental samples with the use of different analytical techniques

Sample	Sampling site	Concentration range	Analytical technique	Reference
Sediment	Arnous River (downstream), France	1–10 nmol L^−1^	ICP-MS	Casiot et al. (2011)
	Arnous River (downstream), France	0.2–26.6 nmol L^−1^	ICP-MS	Casiot et al. (2011)
	Arnous River (upstream), France	0.05–0.78 nmol L^−1^	ICP-MS	Casiot et al. (2011)
	Arnous River (upstream), France	0.05–0.83 nmol L^−1^	ICP-MS	Casiot et al. (2011)
	Deule Channel (France)—contaminated sediments near a metal smelter	1.8–1111 μmol kg^−1^	LEAFS	Boughriet et al. (2007)
	Gardon River, France	0.15–0.63 nmol L^−1^	ICP-MS	Casiot et al. (2011)
	Reigous Bay, France	20–235 nmol L^−1^	ICP-MS	Casiot et al. (2011)
	Reigous Bay, France	0.0001–2.6 μmol L^−1^	ICP-MS	Casiot et al. (2011)
	Biwa Lake (Japan)	7.4 ± 0.7 nmol g^−1^	LEAFS	Cheam et al. (1998)
	Buffalo River (USA)	5.5 ± 0.5 nmol g^−1^	LEAFS	Cheam et al. (1998)
	Winter Green Lake (USA)	0.01–0.11 mmol g^−1^	AAS	Cheam (2000)
	Indiana Harbor artificial channel (USA)	6.3 ± 0.4 nmol g^−1^	LEAFS	Cheam et al. (1998)
	Erie Lake (Canada)	0.014–0.053 nmol L^−1^	GFAAS	Lin and Nriagu (1999)
	Humber River (Canada)	2.3 ± 0.4 nmol g^−1^	LEAFS	Cheam et al. (1998)
	Huron Lake (Canada)	0.013–0.088 nmol L^−1^	GFAAS	Lin and Nriagu (1999)
	Lakes near a coal mine (western Canada)	0.001–0.02 μmol g^−1^	LEAFS	Cheam (2000)
	Michigan Lake (Canada)	0.047–0.094 nmol L^−1^	GFAAS	Lin and Nriagu (1999)
	Mine water (western Canada)	0.001–6.5 nmol L^−1^	LEAFS	Cheam (2000)
	Niagara River (Canada)	5.2 ± 0.4 nmol g^−1^	LEAFS	Cheam et al. (1998)
	Ontario Lake (Canada)	0.53–4.2 nmol g^−1^	LEAFS	Borgmann et al. (1998)
	Ontario Lake (Canada)	4.5 ± 0.4 nmol g^−1^	LEAFS	Cheam et al. (1998)
	Ontario Lake (Canada)	0.024–0.034 nmol L^−1^	LEAFS	Cheam et al. (1995)
	Ontario Lake (Canada)	0.026–0.040 nmol L^−1^	LEAFS	Cheam et al. (1995)
	Port Hamilton (Canada)	12.6 ± 1.4 nmol g^−1^	LEAFS	Cheam et al. (1998)
	Port Hamilton (Canada)	0.11–0.18 nmol L^−1^	LEAFS	Cheam et al. (1995)
	Port in Toronto (Canada)	3.0 ± 0.3 nmol g^−1^	LEAFS	Cheam et al. (1998)
	Saint Clair Lake (Canada)	3.1 ± 0.6 nmol g^−1^	LEAFS	Cheam et al. (1998)
	Sudbury (Canada)	2.4 ± 0.1 nmol g^−1^	LEAFS	Cheam et al. (1998)
	Superior Lake (Canada)	4.4–6.8 pmol L^−1^	LEAFS	Cheam et al. (1995)
	Superior Lake (Canada)	0.007 ± 0.001 nmol L^−1^	HR-ICP-MS	Field and Sherrell (2003)
	Tantare Lake (Canada)	38 ± 0.3 pmol L^−1^	ICP-MS	Laforte et al. (2005)
	The Great Lakes (Canada)	0.5–1043 pmol L^−1^	LEAFS	Cheam et al. (1998)
	Vose Lake (Canada)	0.59–1.53 nmol g^−1^	ICP-MS	Laforte et al. (2005)
	Vose Lake (Canada)	5.5 ± 0.4 pmol L^−1^	ICP-MS	Laforte et al. (2005)
	Ovre Skarsion Lake (Sweden)	36–44 pmol L^−1^	ICP-MS	Grahn et al. (2006)
	Listresjon Lake (Sweden)	59 pmol L^−1^	ICP-MS	Grahn et al. (2006)
	Stensjon Lake (Sweden)	22–39 pmol L^−1^	ICP-MS	Grahn et al. (2006)
	Remmarsjon Lake (Sweden)	32–39 pmol L^−1^	ICP-MS	Grahn et al. (2006)
	Tvaringen Lake (Sweden)	24–31 pmol L^−1^	ICP-MS	Grahn et al. (2006)
	Rivers and streams (Poland)	0.0077–0.72 mmol g^−1^	DPASV	Lis et al. (2003)
	Surface waters (Taiwan)	117 ± 1 pmol L^−1^	ICP-MS	Meeravali and Jiang (2011)
	Deep-sea water (Lanmuchang, China)	0.064–5.382 μmol L^−1^	ICP-MS	Xiao et al. (2004)
	Ground water (Lanmuchang, China)	<0.0024 μmol L^−1^	ICP-MS	Xiao et al. (2004)
	Surface water (Yanshang, China)	0.03–0.47 pmol L^−1^	ICP-MS	Xiao et al. (2004)
	Surface water (Lanmuchang, China)	0.24–3.67 μmol L^−1^	ICP-MS	Xiao et al. (2004)
	Spring water (Lanmuchang, China)	0.4–151.7 nmol L^−1^	ICP-MS	Xiao et al. (2004)
	Well water (Lanmuchang, China)	0.049–1.859 μmol L^−1^	ICP-MS	Xiao et al. (2004)
	Pearl River (China)	0.0061–0.0935 μmol g^−1^	ICP-MS	Liu et al. (2010)
	North River (China)	0.0045–0.0158 μmol g^−1^	ICP-MS	Liu et al. (2010)
	Elbe (Germany)	0.0041–0.0095 μmol g^−1^	ICP-MS	Liu et al. (2010)
	Streams sediments (Zn–Pb processing area, Poland)	0.0077–0.7193 μmol g^−1^	DPASV	Lis et al. (2003)
	Rivulet sediment (Zn–Pb processing area, Poland)	0.0367 μmol g^−1^	DPASV	Jakubowska et al. (2007)
	Stream sediment (Zn–Pb processing area, Poland)	0.0073–0.0323 μmol g^−1^	DPASV	Karbowska et al. (2014)
	Bolesław-Bukowno, mining area (Upper Silesia, Poland)	0.0016–0.0685 μmol g^−1^	ICP-MS, ASV	Ospina-Alvarez et al. (2014)
	Tamar estuarine sediments (England)	0.0004–0.0011 μmol g^−1^	ICP-MS	Anagboso et al. (2013)
	Tsunami sediments (Thailand)	0.0019–0.0053 μmol g^−1^	DPASV	Lukaszewski et al. (2012)
Snow and ice	Snow (arctic areas of Canada)	0.015–0.0044 pmol g^−1^	LEAFS	Cheam et al. (1998)
	Surface ice (arctic areas of Canada)	0.0015–0.0055 pmol g^−1^	LEAFS	Cheam et al. (1998)
	Deep-sea ice (arctic areas of Canada)	0.0001–0.0045 pmol g^−1^	LEAFS	Cheam et al. (1998)
	Ellesmere island (arctic areas of Canada)	0.0064–0.0108 pmol g^−1^	ETV-ICP-MS	Baiocchi et al. (1994)
	Antarctica (Terra Nova)	0.0009–0.0022 pmol g^−1^	ETV-ICP-MS	Baiocchi et al. (1994)
Sea water	Pacific Ocean	58–77 pmol kg^−1^	ETV-ICP-MS	Baiocchi et al. (1994)
	Atlantic Ocean	59–80 pmol kg^−1^	ETV-ICP-MS	Baiocchi et al. (1994)
	Ross Sea (Antarctica)	22–25 pmol L^−1^	HR-ICP-MS	Baiocchi et al. (1994)
Air	City center (Zagreb)			
	1998	0–0.09 nmol m^−3^	FAAS	Hrsak et al. (2003)
	1999	0–0.01 nmol m^−3^	FAAS	Hrsak et al. (2003)
	2000	0–0.01 nmol m^−3^	FAAS	Hrsak et al. (2003)
	Residential districts			
	1998	0–0.02 nmol m^−3^	FAAS	Hrsak et al. (2003)
	1999	0–0.03 nmol m^−3^	FAAS	Hrsak et al. (2003)
	2000	0–0.04 nmol m^−3^	FAAS	Hrsak et al. (2003)
Soil	Surface soil (Poland)	0.2–145.8 μmol kg^−1^	DPASV	Lis et al. (2003)
	Deep soil (Poland)	0.1–171.7 μmol kg^−1^	DPASV	Lis et al. (2003)
	Soil contaminated by zinc smelter (Poland)	0.0171–0.1468 μmol g^−1^	ICP-MS	Vaněk et al. (2013)
	Soil reference area (Poland)	0.0010–0.0137 μmol g^−1^	ICP-MS	Vaněk et al. (2013)
	Soil floodplain terraces (Poland)	0.0019–0.0022 μmol g^−1^	DPASV	Jakubowska et al. (2007)
	Mine area (Lanmuchang, China)	0.2–0.6 mmol kg^−1^	ICP-MS	Xiao et al. (2004)
	Alluvial soil (Lanmuchang, China)	0.07–0.3 mmol kg^−1^	ICP-MS	Xiao et al. (2004)
	Intact natural soil (Lanmuchang, China)	7–34 μmol kg^−1^	ICP-MS	Xiao et al. (2004)
	Intact natural soil (Yanshang, China)	4.4–6.8 μmol kg^−1^	ICP-MS	Xiao et al. (2004)
	Soil contaminated with pyrite slag (China)	0.0245–0.0734 μmol g^−1^	ICP-MS	Yang et al. (2005)
	Soil background (China)	0.0079–0.0099 μmol g^−1^	ICP-MS	Yang et al. (2005)
	Arable soils (France)	Median 0.0014 μmol g^−1^	GFAAS	Tremel et al. (1997)
	Czech soil	0.0021–0.0039 μmol g^−1^	ICP-MS	Vaněk et al. (2010a, b)
	Czech sandy soil	0.0029–0.0039 μmol g^−1^	ICP-MS	Vaněk et al. (2015)
	Korean soil—near cement plants	0.0059–0.0632 μmol g^−1^	ICP-OES	Lee et al. (2015)
	Korean soil—near mines	0.0009–0.0053 μmol g^−1^	ICP-OES	Lee et al. (2015)
	Iranian soil, Chelpu catchment area	0.0108–0.0264 μmol g^−1^	ICP-ES	Taheri et al. (2015)
Ores and rocks	Sulfide ores (Lanmuchang, China)	0.5–171.2 mmol kg^−1^	ICP-MS	Xiao et al. (2004)
	Coal (Lanmuchang, China)	0.06–0.22 mmol kg^−1^	ICP-MS	Xiao et al. (2004)
	Secondary materials (Lanmuchang, China)	0.12–5.38 mmol kg^−1^	ICP-MS	Xiao et al. (2004)
	Mine waste (Lanmuchang, China)	0.16–12.72 mmol kg^−1^	ICP-MS	Xiao et al. (2004)
	Crushed field stone (Lanmuchang, China)	0.19–2.40 mmol kg^−1^	ICP-MS	Xiao et al. (2004)
	Gold ores (Yanshang, China)	1–78 μmol kg^−1^	ICP-MS	Xiao et al. (2004)
	Coal (Yanshang, China)	1.5–41.1 μmol kg^−1^	ICP-MS	Xiao et al. (2004)
	Magmatic rock (France)	0.0016–0.0083 μmol g^−1^	GFAAS	Tremel et al. (1997)
	Metamorphic rock (France)	0.0013–0.0049 μmol g^−1^	GFAAS	Tremel et al. (1997)
	Clastic rock (France)	0.0002–0.0043 μmol g^−1^	GFAAS	Tremel et al. (1997)
	Calcareous rock (France)	0.0005–0.1057 μmol g^−1^	GFAAS	Tremel et al. (1997)
	Sinemurian limestone (France)	0.0362–0.2691 μmol g^−1^	GFAAS	Tremel et al. (1997)
	Carixian marls (France)	0.0147–0.0167 μmol g^−1^	GFAAS	Tremel et al. (1997)
	Composite rock (France)	0.0002–0.0025 μmol g^−1^	GFAAS	Tremel et al. (1997)
	Alluvial rock (France)	0.0017–0.0036 μmol g^−1^	GFAAS	Tremel et al. (1997)
	Floodplain sands, gravel, and silt (Poland)	0.0022–0.0092 μmol g^−1^	DPASV	Lis et al. (2003)
	Slope wash sands and loams (Poland)	0.0007–0.1717 μmol g^−1^	DPASV	Lis et al. (2003)
	Loesses (Poland)	0.0009–0.0049 μmol g^−1^	DPASV	Lis et al. (2003)
	Glaciofluvial sands and gravel (Poland)	0.0001–0.0113 μmol g^−1^	DPASV	Lis et al. (2003)
	Dolomites, ore-bearing dolomites, limestones, and marls (Poland)	0.0021–0.0298 μmol g^−1^	DPASV	Lis et al. (2003)
	Dolomites (Poland)	0.0014–0.0092 μmol g^−1^	DPASV	Karbowska et al. (2014)
	Ore-bearing dolomites (Poland)	0.0057–0.0396 μmol g^−1^	DPASV	Karbowska et al. (2014)
	Belgian Zn–Pb vein deposits	0.1468–30.8264 μmol g^−1^	X-ray fluorescence, XRF	Duchesne et al. (1983)
	Dumps of Zn–Pb processing (Poland)	0.0338–0.1458 μmol g^−1^	DPASV	Lis et al. (2003)
	Galena concentrate (Poland)	0.0308–0.0357 μmol g^−1^	DPASV	Karbowska et al. (2014)
	Blende concentrate (Poland)	0.0298–0.0494 μmol g^−1^	DPASV	Karbowska et al. (2014)
	Crude Zn–Pb ores (Poland)	0.0091–0.0100 μmol g^−1^	DPASV	Karbowska et al. (2014)
	Carbonate rock (Erzmatt, Switzerland)	0.4893–4.8931 μmol g^−1^	ICP-MS	Voegelin et al. (2015)
Wastewater	Bolesław-Bukowno, mining area (Upper Silesia, Poland)	0.2447 ± 0.0098 μmol L^−1^ (9 ± 1 % as Tl III) (91 % as Tl I)	HPLC with ICP-MS	Ospina-Alvarez et al. (2015)
Volcanic ashes	Iceland (2010) Eruption of *Eyjafjallajökull*	0.0023 ± 0.0002 μmol g^−1^	DPASV	Karbowska and Zembrzuski (2016)
White mustard (*Sinapis alba*)	Czech Republic (central part)	0.1908 μmol g^−1^ (in stem) 0.1028 μmol g^−1^ (in leaf) 0.0636 μmol g^−1^ (in root), DW	ICP-MS	Groesslova et al. (2015)
Green cabbage (*Brassica oleracea* L. var. *capitata* L.)	Guizhou, China	0.4942–0.9395 μmol g^−1^ in the leaves, DW	ICP-MS	Ning et al. (2015)
Urine	Population: pregnant woman Location: Hubei, China	Median 0.0016 μmol L^−1^ (0.0027 μmol g^−1^ in creatinine)	ICP-MS	Xia et al. (2016)
	Population: pregnant woman Location: Spain	Median 0.0009 μmol g^−1^ in creatinine	ICP-MS	Fort et al. (2014)
	Population: opioid addicts Location: Mashhad, Iran	0.0–1.693 μmol L^−1^ Median 0.0685 μmol L^−1^	GFAAS	Ghaderi et al. (2015)

### Thallium in plants

The presence of contaminants comprising thallium in air results in its absorption by plants—a process, which is further enhanced by high thallium content in soil (Groesslova et al. 2015; Małuszyński 2009).

The natural content of thallium in plants is usually at approx. 0.05 mg kg^−1^ (Krasnodębska Ostręga and Golimowski 2008). Samples of clovers collected from uncontaminated regions of Poland contained from 0.008 to 0.01 mg kg^−1^ of thallium, in case of grasses the concentration ranged from 0.02 to 0.6 mg kg^−1^, for vegetables the values reached 0.02–0.3 mg kg^−1^, whereas fungi contained up to 5.5 mg kg^−1^ of thallium (Kicińska 2009; Kabata Pendias and Pendias 1999; Małuszyński 2009). In Poland, the highest emissions of thallium have been recorded in the vicinity of a Zn–Pb smelting and mining complex in the Bukowno-Olkusz region between the towns of Katowice and Cracow. Plant samples (plant foliage) of birch originating from this region contained from 9.4 to 12.6 mg kg^−1^ of thallium in buds and approx. 18.5 mg kg^−1^ in the leaves. Grasses contained 25.5 mg kg^−1^ of thallium on the average. Juices collected from birch trunks contained from 89 to 145 μg L^−1^ of thallium. Plants of the *Brassica* genus display a notable tendency to accumulate thallium. Alleviated levels of this element were found in cabbage, curly kale and oilseed rape (Asami et al. 1996). It was established that the concentration of thallium in cabbage plants is increased with the decrease of soil pH value (Dmowski et al. 2002).

### Thallium in aquatic and terrestrial animals

Thallium concentrations measured in phytoplankton and macrophytes span a large range of values and depend on the type of organism (Twiss et al. 2004), the exposure duration (Kwan and Smith 1991), the aqueous concentration of Tl (Kwan and Smith 1988) and the concentration of K^+^ in the exposure medium (Twiss et al. 2004; Hassler et al. 2007), as well as ambient pH.

Studies regarding sea fish belonging to the salmon family (*Salvelinus namaycush*) from central Pacific Ocean revealed that the concentration of thallium in their body reached 0.2–12 nmol g^−1^ (Lin et al. 2001; Couture et al. 2011). Analysis of alpine trout (*Salvelinus alpinus*) tissues originating from Lake Hazen (Ellesmere Island, Nunavut, Kanada) showed a broad range of thallium concentrations in its muscle tissue (0.07 to 0.61 nmol g^−1^) (Gantner et al. 2009). Thallium concentrations in muscle tissue of northern pike (*Esox lucius*) originating from lakes subjected to uranium milling wastewater discharge were four to five times higher compared to concentrations of thallium in tissues of fish originating from non-contaminated lakes in the same area (Kelly and Janz 2009; Gantner et al. 2009). The highest thallium concentrations were found in muscle tissue of fish collected from aqueous environments in the vicinity of contaminated areas, reaching from 470 (Palermo et al. 1983) to 575 nmol g^−1^ (Zitko et al. 1975). The lowest dissolved thallium concentration for which toxic effects have been reported is 0.15 μmol L^−1^ for juvenile Atlantic salmon (Zitko et al. 1975). Lethal thallium concentrations for 50 % mortality ranging from 20.9 to 294 μmol L^−1^ were reported for fish species such as roach, perch and rainbow trout (Pickard et al. 2001).

Studies regarding animals in the Bukowno-Olkusz region between the towns of Katowice and Cracow (Poland) indicate that the spawn of amphibians may be a very important source of thallium contamination for predators. From among all tissues of the Bukowno adult toads, the livers have shown the highest accumulation of thallium (mean 3.98 mg kg^−1^ dw and maximum value 18.63 mg kg^−1^ dw). For as many as 96.5 % of livers, concentrations exceeded 1.0 mg kg^−1^ dw which is treated as indicative of poisoning (Dmowski et al. 2015).

Thallium accumulation was examined in the liver and kidney of five species of dabbling ducks and three species of diving ducks in Japan (Mochizuki et al. 2005). Organ concentrations ranged between 0.0049 and 0.14 μmol g^−1^ dry weight and were about four times higher in dabbling compared to diving ducks. This difference in Tl concentrations could be due to differences in Tl concentrations in their prey (invertebrates and small fish) since dabbling 11 ducks feeding in shallow areas whereas diving ducks collect food in deeper parts of the same water bodies. (Couture et al. 2011).

Typical concentrations in animal muscle tissue amount to 0.74–110.5 ng g^−1^ (fish), 0.84 ng g^−1^ (rabbit), 1.7 ng g^−1^ (pig) and 0.74 ng g^−1^ (cattle) (Engström et al. 2004; Das et al. 2006; Maluszyński 2009).

### Thallium in human organisms

Increased content of thallium in the human body mainly results from the consumption of contaminated food (vegetables, fish, meat-based products) and drinking water. In case of exposure to air-borne contamination (fly ash), thallium may also enter the human body via lungs. Recent reports indicate that high thallium concentrations may be found in green vegetables, such as cabbage (Ning et al. 2015) or kale (Wallace 2015). A significant increase in thallium content was also observed in case of opioid abusers with mean level of 21 μg L^−1^, compared to 1 μg L^−1^ in the control group (Ghaderi et al. 2015).

In general, it is estimated that a daily diet contains 2 ppb thallium (Wallace 2015). The average content of thallium in the human body measured in US population was approx. 0.1 mg (Lansdown 2013). The concentrations in blood reached 3 μg L^−1^, with reference values being calculated as 0.15–0.63 μg L^−1^ in blood and 0.02–0.34 μg L^−1^ in serum. Fingernails from non-exposed persons showed a threefold higher level of thallium than hair and averaged 0.051 mg kg^−1^. The normal total blood thallium concentration is under 2 μg L^−1^, and concentrations greater than 100 μg L^−1^ are toxic (Lansdown 2013).

Concentration of thallium in human body organs increases in the following order: 0.42–1.5 ng g^−1^ (brain) < 1.5 ng g^−1^ (liver) < 6.1 ng g^−1^ (kidney) < 7–650 ng g^−1^ (hair), <0.6 μg g^−1^ (bone) < 1.2 μg g^−1^ (nail) (Maluszyński 2009). This indicates that Tl accumulates preferentially in peripheral organs, for example nails (Engström et al. 2004; Das et al. 2006).

The majority of thallium, which is not accumulated in the human body, is secreted in urine and, to a lesser extent, in faeces. Therefore, urine tests are considered as the most reliable and accurate ways to measure thallium in human body. Under normal circumstances, thallium in human urine usually does not exceed 1 μg/g of creatinine (Wallace 2015). Thallium can be detected in urine after 1 h and up to 2 months following exposure (Wallace 2015). The measurement is usually based on the addition of a chelating agent or a reagent, which allows for spectrophotometric determination. For example, Nagaraja et al. (2009) described a method which is focused on the oxidation of 3-methyl-2-benzothiazolinone hydrazone hydrochloride (MBTH) by thallium (III) to diazonium cation, which is then treated with imipramine hydrochloride (IPH) in a phosphoric acid medium at room temperature in order to yield a blue-colored product with a maximum absorption at 635 nm.

### Overview of analytical techniques used for determination of thallium in environmental samples

Determination of thallium is a challenging task due to the fact that its concentration in environmental samples may be at a nanogram per gram level or below. Apart from a number of analytical methods used for quantitative determination of the total content of this element, a speciation analysis of different oxidation states for thallium (I) and thallium (III) should also be considered.

### Mass spectrometry-based techniques

Inductively coupled plasma mass spectrometry (ICP-MS) is currently considered as one of the most novel techniques for trace analysis, which is characterized by high sensitivity, precision and selectivity. This technique allows for simultaneous determination of several elements as well as determination of specific isotopes of a given element in complex matrices with a low detection limit (at a pg L^−1^ level) (Szczepaniak 2002). Cao and Xia-jun (2012) determined the concentration of thallium in samples of drinking water and spring water. The detection limit was at 7.0 · 10^−4^ μg L^−1^, and the recovery value ranged from 95 to 102 % with a relative standard deviation value of 0.6 % (Cao and Xia-jun 2012).

Hung-Wei and Shiuh-Jen (2000) used ETV-ICP-MS technique for determination of thallium in sea water samples collected from the area of Kaohsiung (located in southern-western Taiwan). The detection limits were at 0.4–0.5 ng·L^−1^ for all studied samples (Hung-Wei and Shiuh-Jen 2000).

Escudero et al. (2013) developed a rapid and simple method of dispersive liquid-liquid microextraction (DLLME). Initially, a complex of thallium and chloride ions was formed. Tetradecyl(trihexyl)phosphonium chloride (CYPHOS® IL 101) was used in order to obtain an ion pair with the [TlCl_(4)_]^−^, which was later subjected to extraction. In the next step, the phase which contained thallium ions was separated and analyzed with the use of ICP-MS. The dependence of analyte calibration was linear with a correlation coefficient of 0.9989. Under optimal conditions, the detection limit was at 0.4 ng L^−1^. Relative standard deviation (*n* = 10) was at 1 ng mL^−1^. Escudero et al. (2013) confirmed that the method may also be successfully used for a rapid analysis of different thallium forms in water samples (Escudero et al. 2013).

### Atomic absorption spectrometry-based techniques

The FAAS technique is also often employed for the determination of thallium in environmental samples (Griepink et al. 1998; Li et al. 2009). The comparison of results obtained after determination of thallium in different samples with the use of FAAS and ICP-MS techniques is presented in Table 4.

**Table 4:** Use of FAAS and ICP-MS techniques for determination of thallium in water and biological material samples (Li et al. 2009)

Sample	ICP-MS technique	FAAS technique	Relative standard deviation [%]	Recovery [%]
Tap water	0.260 μg L^−1^	0.240 μg L^−1^	3.2	88–102
River water	1.220 μg L^−1^	1.340 μg L^−1^	3.4	91–106
Human hair	0.384 mg kg^−1^	0.395 mg kg^−1^	3.0	90–98
Human nail	0.675 mg kg^−1^	0.668 mg kg^−1^	3.2	88–97

Electrothermal atomic absorption spectrometry (ETAAS) is a well-established technique for monitoring trace amounts of elements in nearly all types of matrices. Zeeman effect electrothermal absorption spectrometry (ZEETAS) was used for determination of thallium in water samples with the use of flotation as a concentration procedure. The detection limit of thallium was at 0.031 μg L^−1^ (Bundalevska et al. 2005).

Cvetkovic et al. (2002) described a simple extraction method during determination of thallium in wine samples with the use of ETAAS. The developed analytical procedure allowed for a 50-fold enrichment and determination of 0.05 μg L^−1^ of thallium in wine. The ETAAS method was also employed for determination of thallium in hair and nails (Asadoulahi et al. 2007) as well as spring water with a detection limit of 0.08 μg L^−1^ (Stafilov and Cundeva 1998).

The GFAAS technique was used for a quantitative analysis of thallium at a nanogram·per gram level in geological materials. The detection limit was at 2 ng g^−1^, the precision of the method ranged from 3.03 to 10.5 % and the relative standard deviation oscillated between 2.1 and 6.7 % (Guo et al. 1992). The results of thallium determination in different environmental samples with the use of GFAAS are presented in Table 5.

**Table 5:** Use of the GFAAS technique for determination of thallium in environmental samples (Asami et al. 1996; Griepink et al. 1988)

Sample	Separation method	Detection limit [ng g^−1^] or [ng mL^−1^]	Interference
Surface water	Extraction	5	Cu
Sea sediments	Extraction	10	Fe, Mo, Re, Au, Sb, Ta
Soil	Extraction	20	Cu, Zn, Pb
Particulate matter	Extraction	3.3	HBr

GFAAS was considered as a technique free of interference and was widely used for determination of thallium in biological samples (Mulkey 1993). Chandler and Scott (1986) conducted studies focused on the determination of thallium in urine samples and achieved a detection limit of 0.1 μg L^−1^ with a relative standard deviation value of 3.5–4.4 %, whereas Schaller et al. (1980) achieved a sensitivity of 0.3 μg L^−1^ for the same matrix. Delves and Shuttler (1991) studied the content of thallium in urine, blood and faecal samples with a detection limit of 5 μg L^−1^ for urine and faecal samples and 10 μg L^−1^ for blood samples.

The hydride generation atomic absorption spectrometry (HGAAS) is characterized by high selectivity (Kumar and Riyazuddin 2010). The detection limit for thallium was at 0.8 ng mL^−1^, which indicates a notable improvement compared to conventional chemical techniques of hydride generation. The proposed method was employed for determination of thallium in a standard solution. The linear range was at 1–250 ng mL^−1^, and relative standard deviation for the method reached a value of 4.2 % (Arbab-Zavar et al. 2009).

The majority of studies focused on the determination of thallium were conducted in water matrices. The results of thallium determination in environmental samples conducted with the use of atomic spectrometry, fluorescent atomic spectrometry and inductively coupled plasma atomic emission spectrometry (ICP-AES) are compared in Table 6.

**Table 6:** Comparison of thallium determination results in environmental samples using different atomic spectrometry techniques

Sample	Preparative method	Analytical method	Detection limit	Reference
Air	Separated in a filter, dissolved in acid	ICP-AES	1 μg g^−1^	Sitting (1985)
Water	Treated with HNO_3_	AAS	0.1 mg L^−1^	NIOSH (1984)
Water	Treated with HNO_3_	AAS	0.1 mg L^−1^	APHA (1985)
Water	Treated with HNO_3_	GFAAS	0.1 μg L^−1^	EPA (1983)
Wastewater	Acid digestion	ICP-AES	40 μg L^−1^	EPA (1983)
Solid waste	Acid digestion	AAS	0.1 mg L^−1^	EPA (1983)
Solid waste	Acid digestion	GFAAS	1 μg L^−1^	EPA (1983)
Solid waste	Acid digestion	ICP-AES	40 μg L^−1^	EPA (1983)

The use of LEAFS for determination of thallium in water originating from port Hamilton (Ontario, Canada) allowed to achieve a detection limit of 0.1 ng L^−1^ (Cheam et al. 1996), whereas samples of spring water collected by Miyazaki et al. contained approx. 1.3 ng mL^−1^ of thallium, as determined by the use of ICP-AES.

### Voltammetry-based techniques

The majority of researchers use the inversion voltammetry techniques (stripping methods) for analysis of thallium content. These techniques offer the best combination of sensitivity and selectivity compared to the abovementioned methods. Voltammetric stripping methods are among the most efficient electrochemical techniques used for trace and speciation analysis. The exceptionally high sensitivity and selectivity of these methods result from the fact that the studied analyte is concentrated prior to determination. The concentration of the analyzed compound is conducted with the use of electrolysis or adsorption on the stationary working electrode. The ‘stripping’ term has been adapted since the concentrated substance is removed from the working electrode (Henze 2003). Compared to conventional polarographic and voltammetric methods, the stripping techniques are superior in terms of sensitivity and their detection limit ranges from 10^−9^ to 10^−11^ mol L^−1^,and in some cases it even reaches 10^−12^ mol L^−1^ (Henze 2003).

The DPASV technique coupled with the flow-injection measuring system (FIA-DPASV), with a detection limit of 0.25 pM, was employed for determination of thallium in soil. The content of thallium in the studied soil samples ranged from 100 to 350 ng g^−1^ (Lukaszewski et al. 2010; Lis et al. 2003).

The results of thallium determination in a wide spectrum of different environmental samples with the use of inversion voltammetry techniques are presented in Table 7.

**Table 7:** Comparison of thallium determination results in different environmental samples with the use of inversion voltammetry techniques (Griepink et al. 1998)

Sample	Electrolyte	Type of electrode used in the voltammetric technique	Electrolysis parameters	Interference	Detection limit [ng∙L^−1^]
Natural water	pH 4.8; EDTA	HMDE—hanging mercury drop electrode	−0.9 V/SCE	Bi, Cu, Sb	0.5
Natural water	pH 4.5; octane EDTA	MFE—mercury-film electrode	−0.8 V/Ag/AgCl	Cd, Pb, Cu	0.011
Sea water	EDTA	MFE—mercury-film electrode	−1.1 V	Cd, Pb	40
Sea water	pH 3.5; KNO_3_	MFE—mercury-film electrode	−0.9 V/SCE	Pb, Cu	0.6
Sea water	KNO_3_/EDTA	MFE—mercury-film electrode	−1.2 V/Ag/AgCl	Cu, Pb, Cd, Zn, Bi, Co, Ni, Sn, Fe	–
Rocks	pH 7–8; citrate EDTA	HMDE—hanging mercury drop electrode	−0.75/SCE	Requires separation	–
Salts	pH 4.5; EDTA	HMDE—hanging mercury drop electrode	−0.6/SCE	Cu, Pb, Cd, Bi, Sb, Sn	200

### Speciation of thallium

Thallium occurs in two oxidation states in the environment: monovalent Tl(I) and trivalent Tl(III). The oxidation state directly influenced the toxicity of thallium—trivalent Tl is approximately 50,000 times more toxic compared to monovalent Tl. Furthermore, Tl(I) may be oxidized to Tl(III) due to the activity of the phytoplankton (Twining et al. 2003). As a result, the toxicity of both species is influenced by their stability, which is associated with the type of sample matrix and the corresponding environmental conditions. It is important to determine the concentration of specific Tl species in environmental and biological samples to properly evaluate the exposure risks. However, the speciation of Tl may be challenging, due to the fact that the species are present at trace concentrations. Hence, the methods used for speciation of Tl should be characterized by very high sensitivity.

Determination of Tl(I) and Tl(III) species was may be carried out in soil samples (Voegelin et al. 2015), wastewater samples (Ospina-Alvarez et al. 2015), plant tissues (Sadowska et al. 2016) and cells (Nowicka et al. 2014). Several reports indicate that the use of ICP-MS allows for credible speciation of Tl. Szopa and Michalski (2015) established that the advantages of this technique include extremely low detection and quantification limits, insignificant interference influence and high precision and repeatability of the determinations. Prior to determination, the separation of Tl(I) and Tl(III) may be achieved by employing a cation exchange guard, ion exchange resins, anion exchange chromatography and size exclusion chromatography (Nolan et al. 2004). A recent study also suggests that the use of anionic surfactants, such as sodium dodecyl sulfate (SDS), may facilitate the separation of thallium species during solid-phase extraction (Biaduń et al. 2016). Yun-Ling et al. (2012) also employed RP-HPLC coupled with ICP-MS for speciation of Tl.

## Summary

Generally, thallium is present in the natural environment at low concentrations. Thallium enters the environment primarily as a result of coal burning and smelting. Air emissions and subsequent depositions of thallium from anthropogenic sources resulted in the increase of its concentrations in areas near industrially relevant objects (smelters, coal combustion power plants, cement plants, etc.). In the vicinity of contaminated areas, elevated concentrations of thallium were found in edible resources, such as vegetables, fruit and tissues of farm animals. This is an issue of major concern, since thallium salts are now considered to be among the most toxic known compounds.

Further studies are required in order to determine other potential sources of thallium in order to limit the negative impact of such contaminants on the environment and human health. Living near hazardous waste sites containing thallium may result in considerably higher exposures. It should also be emphasized that the effects of chronic exposure to low concentrations of thallium are currently unknown. The concentration of thallium in industrial waste, sediments and wastewaters should be strictly monitored, and any by-products containing thallium oxides should be properly secured and labelled.

**Fig. 3:**
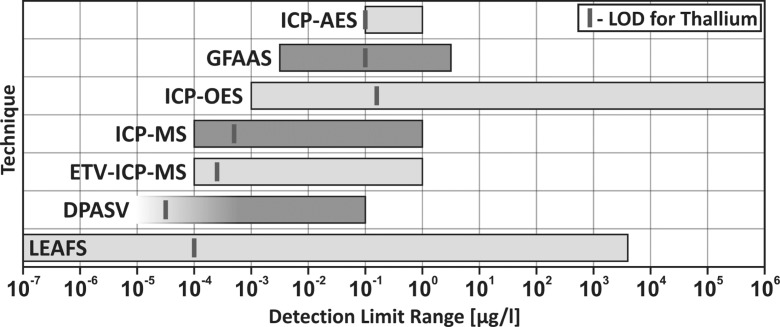
Comparison of thallium LOD values for different analytical methods

A summary of analytical techniques is presented in Fig. 3.

The main benefits of MS-based techniques (e.g. ICP-MS) include the possibility to conduct single and multi-element analyses, a broad range of linearity (which usually covers 4 or 5 orders of magnitude of concentrations of analytes in the sample), no contaminations, high sensitivity and selectivity, simple and rapid analysis and low interference due to matrix effects; their general disadvantages are associated with high analysis cost, the use high purity reagents and the necessity to dilute the samples or digest the matrix.

The main benefits of AAS-based techniques (e.g. FAAS, ETAAS) include an easy and direct method of determination (no pre-treatment is necessary), relatively low analysis costs and low analysis time; their general disadvantages are associated with the susceptibility to matrix effects and the fact that only single elements may be determined. The sensitivity and reproducibility vary between specific methods.

The main benefits of AFS-based techniques (e.g. LEAFS) include the possibility to perform multi-element analyses, high selectivity and sensitivity, a broad range of linearity and low level of interference; their general disadvantages are associated with high costs and analysis time, susceptibility to matrix effects and the fact that the method used for sample preparation or mineralization may notably influence the results.

The main benefits of combined techniques (e.g. FIA-DPASV) include extremely low detection and quantification limits, a marginal influence of interference on the results as well as a notably high precision and repeatability of measurements; their general disadvantages are associated with high apparatus cost and complexity of their operation, which make them less widely available for use in laboratories, and the fact that their use requires a high proficiency with analytical methods and detailed knowledge of instruments.

Selection of an appropriate analytical technique for quantitative and qualitative analysis of thallium depends on the physical state of the sample, its volume, possible decomposition and processing options. However, the sensitivity, detection and quantification range, selectivity and precision should be treated as the main criteria. Selection of an appropriate method for routine analyses should always be based on the analysis of all possible operational factors associated with their use (performance, complexity, time and reagent consumption, type of analyzed sample matrices, etc.).
